# Human mesenchymal stem cells (MSCs) for treatment towards immune- and inflammation-mediated diseases: review of current clinical trials

**DOI:** 10.1186/s12929-016-0289-5

**Published:** 2016-11-04

**Authors:** Li-Tzu Wang, Chiao-Hsuan Ting, Men-Luh Yen, Ko-Jiunn Liu, Huey-Kang Sytwu, Kenneth K. Wu, B. Linju Yen

**Affiliations:** 1 Regenerative Medicine Research Group, Institute of Cellular & System Medicine, National Health Research Institutes (NHRI), 35 Keyan Road, Zhunan, 35053 Taiwan; 2 Graduate Institute of Life Sciences, National Defense Medical Center (NDMC), Taipei, Taiwan; 3 Department of Ob/Gyn, National Taiwan University Hospital & College of Medicine, National Taiwan University, Taipei, Taiwan; 4 National Institute of Cancer Research, NHRI, Tainan, Taiwan; 5 Graduate Institute of Microbiology and Immunology, NDMC, Taipei, Taiwan; 6 Graduate Institute of Basic Medical Sciences, China Medical College, Taichung, Taiwan

**Keywords:** Mesenchymal stem cells, Human, Immunomodulation, Inflammation, Clinical trials, Autoimmune disease, Organ transplantation and rejection, Stem cell therapy

## Abstract

Human mesenchymal stem cells (MSCs) are multilineage somatic progenitor/stem cells that have been shown to possess immunomodulatory properties in recent years. Initially met with much skepticism, MSC immunomodulation has now been well reproduced across tissue sources and species to be clinically relevant. This has opened up the use of these versatile cells for application as 3rd party/allogeneic use in cell replacement/tissue regeneration, as well as for immune- and inflammation-mediated disease entities. Most surprisingly, use of MSCs for in immune-/inflammation-mediated diseases appears to yield more efficacy than for regenerative medicine, since engraftment of the exogenous cell does not appear necessary. In this review, we focus on this non-traditional clinical use of a tissue-specific stem cell, and highlight important findings and trends in this exciting area of stem cell therapy.

## Stem cell therapy for immune- and inflammation-mediated diseases

Stem cells are likely the most promising agent for the treatment of degenerative and ischemic diseases due to their self-renewal and multilineage differentiation capacity. The most exciting aspect of these unique cells is their potential therapeutic impact for regenerative medicine [1, 2]. The best studied type of stem cell is the hematopoietic stem cell (HSC), and transplantation of these tissue-specific stem cells have now become standard-of-care for numerous indications [3]. Over 50 years in the making, the success of HSC transplantation is illustrative of the paradigm for stem cell therapy: replacement and regeneration of pathological endogenous tissue with autologous or 3rd party/allogeneic stem cells. While research in stem cell biology is mainly focused on this goal, an unexpected new avenue of clinical application has emerged for the mesenchymal stem cell (MSC) as an immunotherapeutic agent. A type of somatic progenitor/stem cell, the MSC is capable of multilineage differentiation. However, in recent years, consistent reports on its immunomodulatory properties have opened up the use of these cells for indications other than regenerative medicine. The therapeutic application of MSCs in immune/inflammatory contexts may be more efficacious than traditional indications for regenerative medicine, since engraftment of infused/transplanted stem cells—which have proved surprisingly difficult to achieve [4]—appears not to be necessary for efficacy [5]. In this review, we specifically focus on this non-traditional application of a tissue-specific stem cell, and highlight important findings and trends in this exciting area of stem cell therapy.

## Background: Functional capacity of Mesenchymal stem cells (MSCs)

MSCs were first isolated from the adult bone marrow (BM), and distinguished from marrow hematopoietic cells by their adherent nature in in vitro cell cultures and fibroblastic morphology [6, 7]. The function of BMMSCs was initially thought to be limited to supporting hematopoiesis; indeed, one of the first clinical use of these progenitor/stem cells was to enhance HSC engraftment [8]. Since these early reports, MSCs have been demonstrated to exist in a wide range of adult and fetal organs/tissues [9], and popular sources for isolation other than the BM include adipose tissue, umbilical cord blood, umbilical cord and placenta. In 2006, the International Society for Cellular Therapy (ISCT) established the following unified and minimal criteria to define MSCs [10].

- Plastic-adherent cells
- Expression of the surface markers CD73, CD90 and CD105, but not the hematopoietic markers CD45, CD34, CD14, CD11b, CD19, CD79a or HLA-DR
- Trilineage mesenchymal differentiation capacity into osteoblasts, adipocytes and chondrocytes

In the early 2000’s, reports of immunomodulatory properties in BMMSCs began to emerge [11–13]. While initially met with much skepticism, the reproducibility of these findings using multiple species and disease models along with human case reports established that in vitro cultured MSCs clearly are immunosuppressive and immunomodulatory [14–16]. Moreover, these properties were not limited to MSCs from the BM, but also found with other sources of MSCs, especially fetal sources [17, 18]. Interestingly, despite the increasing number of reports on MSC immune-related functions, the question of why these somatic progenitor/stem cells harbor these properties remain much of a mystery. Regardless of this issue, MSC immunomodulatory functions have greatly expanded the clinical utility of this progenitor/stem cell over other stem cell types, since this allows 3rd party/allogeneic use. Moreover, use of MSCs for immune-/inflammation-mediated disease entities appear to yield more efficacy than for cell replacement/tissue regeneration, since engraftment of the exogenous cell is not necessary. These reasons, along with easily accessible sources for isolation, help explain the popularity of MSC therapy for immune-and inflammation-mediated diseases.

## Clinical status of MSC therapy for immune-/inflammation-mediated diseases

### Disease indications in clinical trials utilizing MSCs

**Fig. 1:**
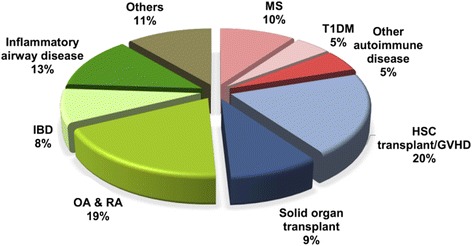
Clinical application of human mesenchymal stem cells (MSCs) for immune- and inflammation-mediated diseases. Graph is a summary of the number of clinical trials using MSC therapy in immune-/inflammation-mediated diseases, as registered on the website https://clinicaltrials.gov/ (accessed April 2016). MS, multiple sclerosis; T1DM, type 1 diabetes mellitus; GVHD, graft versus host disease; OA, osteoarthritis; IBD, inflammatory bowel disease

The capacity of MSCs for multilineage differentiation as well as immunomodulation has meant that these somatic progenitor cells are highly versatile for a wide range of therapeutic applications. Moreover, a number of animal model and translational studies have reported on the capacity of MSCs to home to sites of injury and/or inflammation, thus adding to their attractiveness for clinical use [19]. Indeed, as of April 2016, there were over 500 MSC-related clinical trials registered on the NIH Clinical Trial Database (https://clinicaltrials.gov/). Surprisingly, while the immunomodulatory properties of MSCs have only more recently been identified, nearly half of all registered clinical trials—230 trials or 42 % of all registered trials—are being conducted for immune-/inflammation-mediated diseases (Fig. 1). The main clinical indications within these trials include autoimmune diseases (*n* = 51), organ transplantation and rejection (*n* = 67), and other inflammatory aspects of various diseases (*n* = 112). These trials generally are Phase 1 studies to evaluate safety (*n* = 49 or 21.3 %; 2 Phase 0 trial to establish dosage safety in a small number of subjects), Phase 2 studies to evaluate efficacy (*n* = 53 or 23.0 %), or combined Phase 1/2 studies (*n* = 103 or 44.8 %). A small number of trials are in Phase 3 (*n* = 10 or 4.3 %) or combined Phase 2/3 (*n* = 8 or 3.5 %). There is only one Phase 4 trial to monitor side effects after marketing, and there are 4 trials which did not specify a trial Phase (*n* = 4 or 1.7 %) (Table 1). Trials also differ in terms of the tissue source of MSCs used, with the most frequent reported source being adult BMMSCs (41.2 %). However, other tissue and fetal source MSCs are also popular choices, with 16.3 % of trials using adipose-derived MSCs, and 21.1 % of trials using fetal-source MSCs which includes MSCs isolated from umbilical cord, umbilical cord blood, and placenta (Table 1). While 32.5 % of all trials specify the use of autologous sources, over 50.9 % of trials appear to use allogeneic sources, i.e. trials which use fetal-source MSCs on adult patients. Unspecified donor sources account for approximately 16.7 % of trials. Clearly, the capacity to use allogeneic/3rd party source MSCs greatly contributes to the popularity of this stem cell source. In this review, we will focus our attention on disease indications which have a higher number of clinical trials being conducted.

**Table 1:** MSC clinical trials for immune-related diseases

MSC source	Total %	Total No.	No. of clinical trial phases							
			?	0	1	1 & 2	2	2 & 3	3	4
Unspecified	21.0	49	1	0	9	21	14	3	1	0
Bone marrow	41.2	96	3	1	21^a^	38	25	3	5	0
Adipose tissue	16.3	38	0	1	10	17	7	1	2	0
Umbilical cord	14.2	33	0	0	6^ab^	22	4^c^	0	0	1
Umbilical cord blood	5.6	13	0	0	3	4	3	1	2	0
Placenta	1.3	3	0	0	2^b^	0	1^c^	0	0	0
Menstrual Blood	0.4	1	0	0	0	1	0	0	0	0
Total No. of clinical trial phases		230	4	2	49^d^	103	53^e^	8	10	1
Total % of clinical trial phases			1.7	0.9	21.3	44.8	23.0	3.5	4.3	0.4

^abc^MSCs are applied to the same clinical trials

^de^Number of total clinical trial phase 1 and phase 2 withdraw duplicated MSC source number in the same clinical trials

### Mechanisms of human BM and other tissue source MSC immunomodulation

Since the first studies demonstrating immunomodulation by MSCs, there have been significant advances in understanding mechanisms involved in these properties, including interactions with specific leukocyte populations [16, 20]. MSC modulation of CD4 T lymphocyte populations has been best studied, with most reports demonstrating that secreted factors such as transforming growth factor β1 (TGF-β1) and prostaglandin E_2_ (PGE_2_) being involved in inhibiting T cell proliferation [21]. In addition, MSCs can modulate T lymphocyte fate, polarizing naïve CD4 towards a regulatory T cell (Treg) phenotype and shifting the cytokine profile from a T helper cell type 1 (Th1)—in which high levels of interferon-γ (IFN-γ) and tumor necrosis factor-α (TNF-α) are secreted—to a Th2 milieu [22]. MSCs can suppress the cytotoxic activity of CD8 cytotoxic T cells [23, 24] as well as natural killer cells (NK) [25], and can also interfere with B cell maturation and antibody production [26, 27]. In addition to interacting with adaptive and innate lymphocyte populations, MSCs have also been shown to modulate the differentiation, expansion, and/or function of myeloid cells towards more immunosuppressive and immunomodulatory phenotypes. These interactions include myeloid populations ranging from monocytes [28, 29], dendritic cells (DCs) [30, 31], macrophages [32, 33], and myeloid-derived suppressor cells (MDSCs) [34]. Most recently, there is also data showing modulation of granulocytes by BM and placental MSCs [35, 36]. In studies using animal disease models, efficacy was especially prominent in experimental autoimmune encephalomyelitis (EAE) and moderate for collagen-induced arthritis (CIA), which are models for multiple sclerosis (MS) and rheumatoid arthritis (RA), respectively [20, 37]; an early human case report demonstrated efficacy of allogeneic BMMSCs towards graft-versus-host disease (GVHD) [14].

**Fig. 2:**
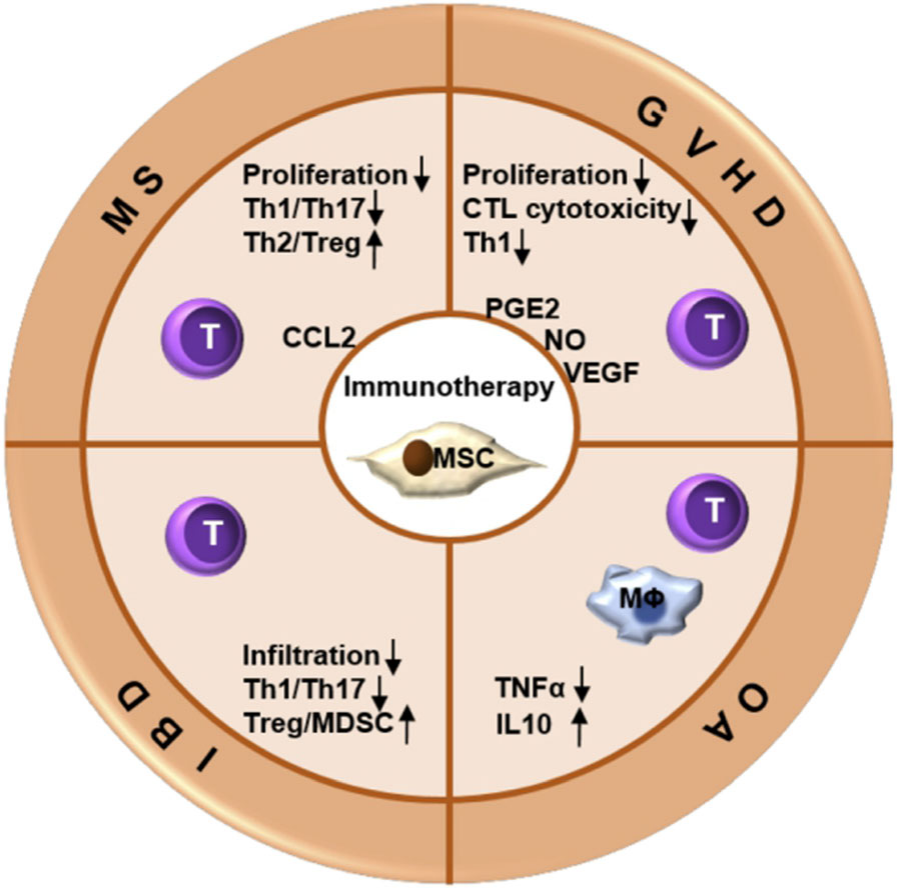
MSC-derived paracrine factors mediate immunomodulatory functions, particularly towards T lymphocytes, in preclinical animal studies of various immune-and inflammation-mediated diseases

In correlation to animal studies and human case reports, the most common immune-/inflammation-mediated indications in MSC clinical trials were for GVHD (*n* = 46), osteoarthritis (OA; *n* = 38), inflammatory airway diseases (*n* = 29), MS (*n* = 23), and solid organ transplant rejection (n =21). The majority of trials are still ongoing, with less than 7 % of trials with published results; these published reports have been for clinical trials on MS [38], GVHD [39–41], OA [42–46], inflammatory bowel disease (IBD) [47, 48] and various pulmonary inflammatory diseases [49–51]. In this review, therefore, we will discuss the possible mechanisms and clinical efficacy of MSC treatment for these particular indications (Fig. 2).

## State of MSC clinical research in specific immune-/inflammation-mediated diseases

### Graft-versus-host disease (GVHD)

The most successful therapeutic application using stem cells has been with HSCs [52]. These tissue-specific stem cells can be isolated from adult BM, cord blood, or mobilized to peripheral blood, and represent a life-saving treatment for patients with hematopoietic malignancies and genetic diseases, including hereditary anemia and immunodeficiencies. Either autologous or matched allogeneic/3rd-party HSC transplantation may be performed depending on the clinical scenario. With allogeneic/3rd-party HSC transplantation, immunosuppression is necessary. But despite immunosuppressant therapy, immune rejection in the form of GVHD is still a major cause of morbidity and mortality, occurring in 30 ~ 40 % of allogeneic HSC transplantations [53]. The presence of allo-reactive donor lymphocytes is the crucial reason for GVHD, which are responsible for the inflammatory injury to multiple organs, most commonly the skin, gastrointestinal tract, and liver [54, 55]. The clinical application of MSCs for GVHD developed more rapidly than for any other type of immune-/inflammation-mediated diseases, likely due in large part to a case report in which a pediatric patient with severe GVHD was infused with haploindentical BMMSCs with dramatic therapeutic effect [14]. The scientific basis for this case largely rested on a few human in vitro report showing allogeneic BMMSCs suppressing lymphocyte proliferation and effector functions [11–13], along with clinical safety data from MSC-HSC co-transplantation engraftment trials [8]. In this case report, the patient was a 9 year-old boy with acute lymphoblastic leukemia post-allogeneic HSC transplantation. Despite being on multiple immunosuppressants including two types of corticosteroids, infliximab + daclizumab, as well as cyclosporin, the patient developed severe acute GVHD which lead to the inability to eat by day 24 post-transplantation. Haploidentical BMMSCs from his mother—a readily available donor—was infused at 2 × 10^6^ cells/kg weight, and dramatic decreases in GVHD symptomatology could be seen within a week of MSC infusion. The patient eventually required a 2nd infusion of MSCs at a lower dose of 1 × 10^6^ cells/kg, which along with low levels of immunosuppression (predinosolone + cyclosporine) resolved the GVHD and allowed for the patient to be alive and well many years post-HSC transplantation. Based on this one successful case report, numerous clinical trials for GVHD using autologous, haploidentical, and/or unmatched MSCs have subsequently been conducted. Among completed trials with published reports are two large-scale multicenter Phase 2 studies for treatment of steroid-resistant, severe acute GVHD, both of which showed striking efficacy [56, 57]. Smaller trials on other related complications have also been published: refractory cytopenias [58] and attenuated dry eye in patients with chronic GVHD [59, 60]. Currently, there are 46 registered trials of MSCs for GVHD and related complications. Most of these trials are Phase 2 (*n* = 20) or combined Phase 1/2 trials (*n* = 15), whereas a small number are Phase 1 (*n* = 3), Phase 3 (*n* = 3), combined Phase 2/3 (n =3), or undefined trials (*n* = 2). BM is the major source of MSCs in GVHD trials (*n* = 22), with a few trials utilizing MSCs from other sources including adipose tissues (*n* = 3), umbilical cord (*n* = 1) and umbilical cord blood (*n* = 3). 17 trials did not specify the source of MSC used. A few currently registered trials have published results, and all demonstrate safety of MSC use in GVHD patients as well as some efficacy [39–41].

Despite the promising results of several MSC trials for GVHD treatment, this trend was not consistently seen in all trials [61]. A recent meta-analysis revealed much heterogeneity in conducted trials both on the patient end—which include differences between pediatric vs. adult patients, type of HSC transplanted (BM, peripheral blood, or cord blood)—as well as with the MSCs utilized [62]. A striking difference in published trials conducted in Europe (with generally positive results) compared to North America (with more equivocal results) has been in the MSCs used in terms of culture conditions, passage number, and whether cryopreservation was involved [63, 64]. Adding to the problem may be the fact that detailed mechanisms on acute GVHD are still somewhat unclear, and even more so for chronic GVHD [65]. Thus, there is continued research using mouse and other animal models to further understand the pathophysiology of these diseases. A number of mouse GVHD models—including humanized mouse models—have been developed, and the infusion of mouse and human BMMSCs have generally demonstrated efficacy against the disease by suppressing donor leukocyte inflammatory responses [66–68]. MSC factors involved include PGE_2_ [69] and nitric oxide (NO) [70]; and effects can be enhanced with pretreatment of IFN-γ to the MSCs [68]. Animal model studies also demonstrate that sources of MSCs other than BMMSCs may also ameliorate GVHD, and may involve vascular endothelial growth factor (VEGF), PGE_2_, and TGF-β [71–74]. One advantage of MSC immunodulation compared with immunosuppressant drug therapy may be the capacity of MSCs to inhibit GVHD processes while preserving graft-versus-leukemia (GVL) effects, a process thought to eliminate primary or secondary cancer/tumor formation [69]. This may be due to the fact that MSCs—regardless of source—highly expand Tregs [18, 75], a CD4 population now thought to be critical for simultaneously inhibiting GVHD without compromising GVL responses [76]. Clearly, MSCs have strong potential as therapeutic agents for GVHD, but detailed tailoring of patient population and stringent MSC processing criteria are necessary to deliver consistent and reproducible results.

### Multiple sclerosis (MS)

MS is an inflammatory and demyelinating disorder of the central nerve system (CNS), and current studies have found that both Th1 and interleukin-17A (IL-17A)-secreting CD4 (Th17) lymphocytes are involved in the pathogenesis of this autoimmune disease [77, 78]. MS has long been known to be a CD4 T-cell mediated autoimmune disease that targets myelin-based protein (MBP), a protein found specifically in myelin sheaths [79]. The resulting demyelination leads to neuronal damage and conduction impairment, which explains the ‘waxing and waning’ nature of the disease. Symptoms are progressive and debilitating, and include blurred vision, blindness, partial or total paralysis, memory and cognitive deficits [80]. Currently without cure, MS is the most common autoimmune disease of the CNS and as of 2013, an estimated 2.3 million people are affected with the disease, with women twice as likely as men to be affected [81].

One of the best animal models for MS is EAE in mice and using this model, treatment with MSCs has demonstrated strong therapeutic effects [37, 82]. Intravenous administration of either mouse or human MSCs can be detected in the lymphoid organs and demyelinating regions of EAE mice, and results in amelioration of inflammation as well as symptoms and disease course [82, 83]. MSC treatment suppresses auto-reactive Th1/Th17 proliferation and infiltration in both in vitro and in vivo studies [82, 84, 85]. Other reports show that MSC treatment increases accumulation of Th2 cytokines—IL-4 and IL-5—and generation of Treg in vivo, both of which help reduce EAE symptomatology [83, 86]. Molecular mechanisms by which MSCs polarize CD4 T cells in EAE models include via indoleamine-2,3-dioxygenase (IDO) and monocyte chemoattractant protein-1/CC chemokine ligand 2 (MCP-1/CCL2) [87]. Interestingly, an in vitro human study found that while MSCs can effectively inhibit proliferation and IL-2 production by T cells isolated from MS patients as well as normal controls, T cells of MS patients still produce higher levels of IL-2 compared to normal control T cells, demonstrating the inherent pathological immune responses in these patients [88]. Based on these and many other preclinical studies demonstrating MSC therapeutic efficacy in animal disease models, these stem/progenitor cells were considered as strong candidates for treatment of patients with MS.

To date, there are 23 registered clinical trials using MSCs for treatment of MS, with 4 in Phase 1, 4 in Phase 2, and 15 as combined Phase 1/2. Sources of MSCs used in these trials are from the BM (*n* = 11), umbilical cord (*n* = 4) and adipose tissue (n =2), with 6 studies using unspecified sources. In general, the number of MSCs transplanted is approximately 2 × 10^6^ cells/kg given either intravenously or intrathecally. One clinical trial has published results on determination of safety and efficacy of intravenously administration of autologous BM-MSCs for MS patients [38]. This Phase 2A trial, which included 10 MS patients and 8 healthy controls, demonstrated that the treatment was safe. While efficacy was difficult to evaluate, a few outcome parameters—mainly of optic nerve-based measures—demonstrated statistically significant or near significant improvement. The importance of this trial was also to establish detailed trial design and clinical efficacy measures for MSC therapy in MS. Resolution of these critical issues will help to pave the way for use of MSCs, which is one of the most novel methods of treating MS, in this incurable CNS disease.

### Joint diseases: Osteoarthritis (OA) & Rheumatoid Arthritis (RA)

MSCs are an important therapeutic option for joint disease, since cartilage does not regenerate and these progenitor/stem cells are the endogenous progenitor for this tissue. Two major joint disease entities have been targeted for MSC treatment: OA and RA. OA is the most common joint disorder which is due to gradual deterioration of joint cartilage from ‘wear-and-tear.’ This subsequently induces an immune response with further resultant damage to joints [89]. Since cartilage does not regenerate, OA is a progressive and irreversible condition, with the incidence increasing with age and body weight. While immune injury is not the causative reason for OA, by the time patients seek medical help due to pain and joint stiffness, inflammation is well underway. Moreover, inhibition of the vicious cycle of cartilage destruction and immune attack is necessary in order for joint repair to occur. As such, MSCs are particularly suited for use in OA, since cartilage regeneration and immunosuppression can be achieved simultaneously [90]. Indeed, both small and large animal studies demonstrate that MSCs decrease inflammation in OA and allow for cartilage repair [91–93]. Currently, there are 38 clinical trials registered, with 9 in Phase 1, 16 in joint-Phase 1/2, and 8 in Phase 2. Not surprisingly, more than 18 % of studies have published results on the safety and efficacy of MSCs for OA treatment [42–46, 94]. Overall, these studies demonstrate quite positive results regarding improvement in symptomatology—including pain—and joint repair as seen by cartilage regeneration.

While translational and clinical data are generally positive for MSC therapy in OA, surprisingly this is not the case for RA. To date, there are only 5 clinical trials utilizing MSCs for RA treatment registered, with 1 trial in Phase 1, 3 in Phase 1/2 and 1 in Phase 2/3; no trials have yet published results. Unlike OA, RA is an autoimmune disease with a well-established animal model being the CIA model, in which autoimmune joint disease can be reproduced in rodent models [95]. Even with animal models, there are discrepant results with regards to MSC therapeutic effects [20, 96]. Clearly, there are detailed mechanistic differences between RA and OA which still need to be resolved, and may likely explain the therapeutic divergence in MSC therapy for the two joint diseases.

### Inflammatory bowel diseases (IBD)

The etiology and progression of human IBD which includes Crohn’s disease (CD) and ulcerative colitis (UC) are multifactorial, but a critical part of these diseases is the uncontrolled immune responses to intestinal microbes [97]. Both CD and UC are progressively fatal without curative treatment, making MSCs an attractive therapeutic option for these chronic inflammatory diseases.

There are several experimental animal models for IBD, and among the commonly used models are the chemically-induced acute colitis models, with dextran sodium sulfate (DSS) supplemented in drinking water or 2, 4, 6-trinitrobenzene-sulfonate acid (TNBS) administrated by enema [98]. These are also the models in which MSC therapeutic effects were tested on [99, 100]: MSCs can be given by intraperitoneal or intravenous routes, and this can prevent DSS-induced morphological and immunogenic injury of the intestines. Moreover, application of MSCs can specifically reduce Th1 and Th17 responses as well as serum levels of proinflammatory IL-1β, IL-6, IL-17, TNF-α, IFN-γ levels, while enhancing the numbers of Tregs and splenic MDSCs [101, 102]. In TNBS animal models, injection of MSCs resulted in decreased immune cells infiltration and TNF-α expression, but increases of TGF-β levels in sites of injury [103]. To improve the efficacy of MSC treatment for IBD, these progenitor/stem cells have also been coated with antibodies against mucosal addressin cell adhesion moledule-1 (MAdCAM-1) and vascular cell adhesion molecule-1 (VCAM-1), both of which were shown to increase cell delivery to inflamed intestinal regions [104]. Immunosuppression was also enhanced when MSCs was modified with IL-12p40 or IL-37ß [105, 106].

Currently, there are 19 registered clinical trials using MSCs for IBD, with 3 for UC & 16 for CD. With the exception of 4 trials which are in Phase 3, all other trials are in Phase 1 and/or 2. Interestingly, there are already quite a number of published reports regarding treatment of MSCs for fistulas in CD in particular [48]. BM or adipose-derived MSCs were used in these trials, with 2 trials using autologous sources, 11 trials using allogenic source and 2 trials using undefined source. Collectively, a review of 15 trials (some registered at Clinicaltrials.gov but some are not) overwhelmingly demonstrate that MSC therapy is not only safe but therapeutically relevant, with some patients showing durable effects [107]. A very recent trial using allogeneic placenta-derived MSC-like cells (which was not registered) also showed favorable responses [108]. Thus, MSC therapy for IBD—especially CD fistula formation—appears to be safe and a highly viable option.

### Inflammatory airway & pulmonary diseases

**Fig. 3:**
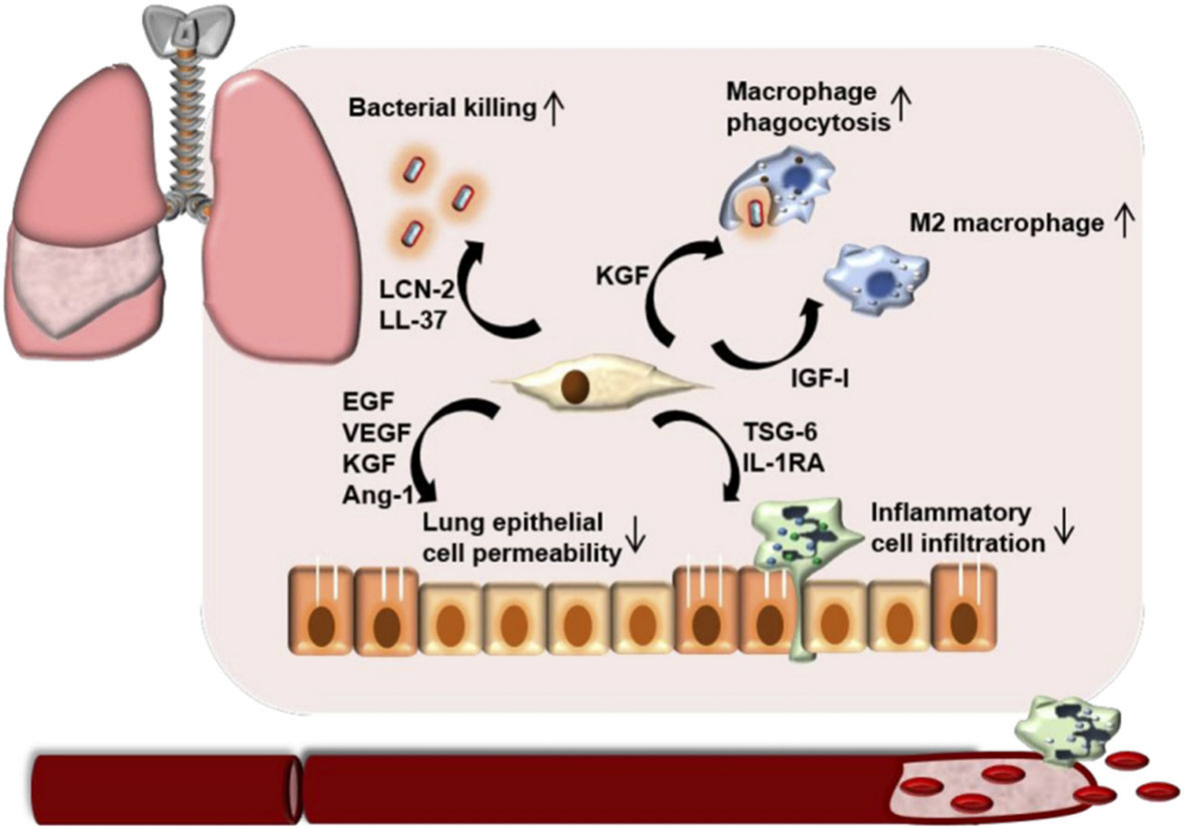
Mechanisms involved in MSC therapy for inflammatory pulmonary diseases based on preclinical animal studies. Immunomodulatory effects include enhancing bacterial clearance by direct killing and enhancement of macrophage phagocytosis; decreasing inflammatory response by modulation of macrophages towards an M2 phenotype and inhibition of neutrophil recruitment; as well as reducing damage to alveolar epithelium

Inflammation is now known to affect many disease processes of the pulmonary system, including obstructive diseases like chronic obstructive lung diseases (COPD) and asthma, as well as restrictive diseases including idiopathic pulmonary fibrosis (IPF) and acute respiratory distress syndrome (ARDS). Whether as cause or consequence, the acute and chronic lung injury found in these diseases invariably involves aberrant immune activity and fibrosis [109, 110]. MSC therapy, indeed most cell therapies, may be particularly suited for use in pulmonary diseases since it has been consistently shown that the overwhelming majority (usually 80 ~ 90 %) of MSCs delivered intravenously—likely the most clinically feasible method for introduction of cellular products—will rapidly reach the lungs [111]. Under conditions of pulmonary inflammation, this percentage increases even further (Fig. 3) [112, 113]. A recent study also suggest that the lung may represent a unique niche for MSCs [114]. Thus, there has been rapid development of using MSCs for a wide range of pulmonary diseases.

Interestingly, while specific inflammatory/immune processes are distinct for pulmonary diseases even within the same classification, i.e. COPD vs. asthma, MSCs have been shown in preclinical studies to impart therapeutic effects despite these pathophysiological differences. In COPD, inflammation driven by alveolar macrophages, cytotoxic T cells, and neutrophils leads to progressive limitations in airflow, with small airway fibrosis and alveolar destruction [115, 116]. In asthma, however, mast cells, eosinophils and Th2 lymphocytes are involved in further aggravation of airway hyperresponsiveness and bronchoconstriction [117]. In rodent models of elastase-induced emphysema or cigarette-induced COPD, MSC infusion reduces lung destruction and aberrant inflammation [118, 119]. MSC-secreted epidermal growth factor (EGF) leads to induction of secretory leukocyte protease inhibitor (SLPI), an inhibitor which protects epithelial tissues from serine protease degradation [120, 121]. Infusion of MSCs in a rat model of cigarette smoke-induced lung injury also results in down-regulation of pro-inflammatory cytokines such as TNF-α, IL-1β, IL-6 and MCP-1/CCL2, and up-regulation of VEGF and TGF-β [122]. In addition, MSC treatment can inhibit cyclooxygenase-2 (COX-2) and COX-2-mediated PGE_2_ production in alveolar macrophages to decrease inflammation [123]. For asthma, in rodent disease models using inhalation of toluene diisocyanate, ovalbumin or cockroach extract, MSC treatment modulated the immune milieu through generation of Tregs and inhibition of Th2 responses [124–126]. Reversal of disease symptomatology along with decreases in Th2 cytokines including IL-4, IL-5, and IL-13, as well as immunoglobulin E (IgE) levels, matrix metalloproteinase deposition, and mucus production were seen [127–129].

Even for fibrotic pulmonary diseases, MSC treatment appears to be efficacious. In fact, one of the earliest studies documenting therapeutic efficacy of MSC infusion was in mouse models of bleomycin-induced lung fibrosis, which is an animal model for IPF [112]. Subsequently, the same group demonstrated that MSC-secreted IL-1 receptor antagonist (IL-1RA) mediated the anti-inflammatory and anti-fibrotic effects [130]. Using the same disease model, infusion of umbilical cord MSCs were also shown to have therapeutic effects [131, 132]. In addition to anti-inflammatory effects, MSC treatment may reduce fibrosis through enhancing the resident lung bronchioalveolar stem cell population for repair and regeneration of healthy lung parenchyma [133]. Such profound effects induced by MSC treatment may account for the rapid push to clinical studies in this field, since about half of the basic and animal studies in this field were published within the past 3 years.

Most interestingly, MSC treatment can have therapeutic results in pneumonia of infectious etiology, especially bacterial pneumonia which clearly elicits intense inflammatory and immune responses. This is somewhat surprising given the strong immunosuppressive effects of MSCs towards effector cell functions. A lethal consequence of infection-induced pneumonia is ARDS, which is a complication with high mortality and morbidity despite medical advancements [134]. Using mice with lung injury induced by lipopolysaccharide, a component of gram-negative bacterial cell wall, delivery of MSCs or MSC-conditioned medium improved tissue damage and survival, which involved MSC-derived factors such as insulin-like growth factor I (IGF-I) and TNF-stimulated gene 6 protein (TSG-6) for generation of anti-inflammatory M2 macrophages and suppression of inflammatory cell infiltration [135–137]. In *Escherichia coli* (*E. coli*)-induced pneumonia rodent models, MSCs improved bacterial clearance by secreting antimicrobial peptide LL-37, antibacterial protein lipocalin 2 (LCN-2) and keratinocyte growth factor (KGF) directly against bacteria or by enhancing macrophage phagocytosis [138–140]. Moreover, administration of BMMSC-conditioned medium-derived microvesicles can also alleviate pulmonary inflammation and injury [141]. MSC treatment for viral pneumonia and subsequent lung injury, on the other hand, may not be as potent, with some reports demonstrating therapeutic effects [142] but not other reports [143, 144]. The dichotomous results of MSC treatment on bacterial compared to viral pneumonia may be due to the fact that MSCs have been shown by multiple studies to modulate neutrophil—the key leukocyte involved in bacterial but not viral infections—life span and functions [35, 36, 145, 146].

To date, 29 clinical studies of using MSCs for pulmonary disorders have been registered. Targeted disease entities include asthma, COPD, ARDS, bronchial pulmonary dysplasia (BPD), and fibrosis (including but not exclusive for IPF), with trials being in Phase 1 (*n* = 14), Phase 2 (*n* = 4), or combined Phase 1/2 (*n* = 11). There are a few published reports of MSC trials for various lung diseases, with the largest published trial being a Phase 2 multicenter study with 62 patients evaluating allogenic BMMSCs for COPD [50]. While safe, the trial did not demonstrate much efficacy. Other published studies are for Phase 1 trials using various tissue-source allogeneic MSCs infused intravenously (except where noted): two trials on ARDS, one using adipose-derived MSCs [147] and one using BMMSCs [51]; one using placental-derived MSCs for IPF [148]; and one using umbilical cord blood MSCs (delivered intratracheally) for preterm BPD [49]. All three reports showed safety of MSC infusion, but efficacy was weak at best. The strong evidence shown in preclinical animal studies does not seem to be replicated in human trials so far, and this may be a consequence of the diversity of lung diseases targeted, as well as the fact that multiple tissue source of MSCs were used. In addition, whether differences in MSC tissue source affect homing capacity is also a critical issue. Thus, careful selection of patient populations and more research into whether tissue-specific MSCs harbor distinct therapeutic effects are warranted.

## Conclusion

The immunomodulatory properties of MSCs have become increasingly relevant for clinical use. Based on hundreds of clinical trials, the safety of this therapy appears clear; less certain is the efficacy of such cell therapy. The overwhelming positive results seen in preclinical animal studies have largely not yet translated into clinical efficacy. Clearly, there is still much to learn and optimize with regards to the in vivo interactions of MSCs in human pathological states. As we improve our understanding on the mechanistic properties of MSC immunomodulation, we also need to clarify pathophysiological details and subsets within disease entities to better tailor MSC therapy. One important aspect is to delineate tissue-specific functional differences in MSCs from difference sources; the current ISCT standardization does not include immune-related functional tests or more detailed molecular validation. In addition, standardization of in vitro culture protocols with stringent criteria for testing of functional parameters is necessary as well. While there is clearly much still to do in this field, it must be remembered that even for HSC transplantation—a clinically established treatment modality—continued evolution in improving engraftment and decreasing complications is still ongoing. Nevertheless, based on current development and results, the tremendous potential of MSC therapy can be expected in the near future to achieve clinical relevance.

## Abbreviations

ARDS: Acute respiratory distress syndrome
BM: Bone marrow
BPD: Bronchial pulmonary dysplasia
CCL2: CC chemokine ligand 2
CD: Crohn’s disease
CIA: Collagen-induced arthritis
CNS: Central nerve system
COPD: Chronic obstructive lung diseases
COX2: Cyclooxygenase-2
DC: Dendritic cell
DSS: Dextran sodium sulfate
E. coli: Escherichia coli
EAE: Experimental autoimmune encephalomyelitis
EGF: Epidermal growth factor
GVHD: Graft-versus-host disease
GVL: Graft-versus-leukemia
HSC: Hematopoietic stem cell
IBD: Inflammatory bowel disease
IDO: Indoleamine-2, 3-dioxygenase
IFN-γ: Interferon-γ
IgE: Immunoglobulin E
IGF-I: Insulin-like growth factor I
IL: Interleukin
IL-1RA: IL-1 receptor antagonist
IPF: Idiopathic pulmonary fibrosis
ISCT: International society for cellular therapy
KGF: Keratinocyte growth factor
LCN-2: Antibacterial protein lipocalin 2
MAdCAM-1: Mucosal addressin cell adhesion moledule-1
MBP: Myelin-based protein
MCP-1: Monocyte chemoattractant protein-1
MDSC: Myeloid-derived suppressor cell
MS: Multiple sclerosis
MSC: Mesenchymal stem cell
NK: Natural killer cell
NO: Nitric oxide
OA: Osteoarthritis
PGE2: Prostaglandin E2
RA: Rheumatoid arthritis
SLPI: Secretory leukocyte protease inhibitor
T1DM: Type 1 diabetes mellitus
TGF-β1: Transforming growth factor β1
Th: T helper cell type
TNBS: 2, 4, 6-Trinitrobenzene-sulfonate acid
TNF-α: Tumor necrosis factor-α
Treg: Regulatory T cell
TSG-6: TNF-stimulated gene 6 protein
UC: Ulcerative colitis
VCAM-1: Vascular cell adhesion molecule-1
VEGF: Vascular endothelial growth factor
